# Essentials of Medical Electricity

**Published:** 1892-12

**Authors:** 


*Essentials of Medical Electricity.* By D. D. STEWART, M.D., and E. S. LAWRANCE, M.D. Pp. 158. Philadelphia: W. B. SAUNDERS. 1892.

This book purports to put the most important points of the subject before "the undergraduates in such a manner that they may form a general idea of the diagnostic and therapeutic value of electricity." This object has been fairly attained, and, so far as it goes, the book is a good one, and likely to be serviceable as a short practical aid in clinical work. Perhaps the authors are somewhat inclined to speak too positively as to the value of electricity in diseases in which its curative effects are, to say the least, problematical.

